# Supplementary material for: MetaRibo-Seq measures translation in microbiomes
Source: Nat Commun. 2020 Jun 29;11:3268. doi: 10.1038/s41467-020-17081-z (PMC7324362; doi:10.1038/s41467-020-17081-z)
Supplement: Supplementary file 10 — Supplementary Data 7 [file 41467_2020_17081_MOESM10_ESM.zip › File2/Confidence_High_Taxonomy/265971_out.krona.html]

Javascript must be enabled to view this page.

members
magnitude
magnitudeUnassigned
count
unassigned
taxon
rank

265971\_out

24

2
24
superkingdom

phylum
1239
24

class
24
186801

order
186802
24

family
24
186803

2
33042
24
genus

SRS1041091\_contig\_number\_contig-100\_15405.90719SRS146764\_contig\_number\_contig-100\_33186.87287


SRS019068\_contig\_number\_72559SRS020869\_contig\_number\_contig-100\_5984.157829SRS024132\_contig\_number\_contig-100\_9498.214074SRS024435\_contig\_number\_contig-100\_200.147259SRS042966\_contig\_number\_14323SRS045713\_contig\_number\_8222SRS048164\_contig\_number\_contig-100\_26368.66182SRS049712\_contig\_number\_15418SRS049959\_contig\_number\_42372SRS050422\_contig\_number\_contig-100\_9868.139806SRS053214\_contig\_number\_614SRS053356\_contig\_number\_41500SRS054956\_contig\_number\_8046SRS063518\_contig\_number\_21262SRS077730\_contig\_number\_1194SRS144537\_contig\_number\_contig-100\_4167.207426SRS146813\_contig\_number\_2893SRS147346\_contig\_number\_27660SRS147919\_contig\_number\_21202SRS148159\_contig\_number\_869SRS148721\_contig\_number\_26761SRS149879\_contig\_number\_contig-100\_905.140624
species
410072
22
